# Current status and future perspectives in targeted therapy of *NPM1*-mutated AML

**DOI:** 10.1038/s41375-022-01666-2

**Published:** 2022-08-25

**Authors:** Roberta Ranieri, Giulia Pianigiani, Sofia Sciabolacci, Vincenzo Maria Perriello, Andrea Marra, Valeria Cardinali, Sara Pierangeli, Francesca Milano, Ilaria Gionfriddo, Lorenzo Brunetti, Maria Paola Martelli, Brunangelo Falini

**Affiliations:** 1 Section of Hematology, Department of Medicine and Surgery, Center for Hemato-Oncological Research (CREO), University of Perugia, Perugia, Italy; 2 Department of Molecular and Clinical Sciences, Università Politecnica delle Marche, Ancona, Italy

**Keywords:** Acute myeloid leukaemia, Drug development

## Abstract

Nucleophosmin 1 (NPM1) is a nucleus-cytoplasmic shuttling protein which is predominantly located in the nucleolus and exerts multiple functions, including regulation of centrosome duplication, ribosome biogenesis and export, histone assembly, maintenance of genomic stability and response to nucleolar stress. *NPM1* mutations are the most common genetic alteration in acute myeloid leukemia (AML), detected in about 30–35% of adult AML and more than 50% of AML with normal karyotype. Because of its peculiar molecular and clinico-pathological features, including aberrant cytoplasmic dislocation of the NPM1 mutant and wild-type proteins, lack of involvement in driving clonal hematopoiesis, mutual exclusion with recurrent cytogenetic abnormalities, association with unique gene expression and micro-RNA profiles and high stability at relapse, *NPM1*-mutated AML is regarded as a distinct genetic entity in the World Health Organization (WHO) classification of hematopoietic malignancies. Starting from the structure and functions of NPM1, we provide an overview of the potential targeted therapies against *NPM1*-mutated AML and discuss strategies aimed at interfering with the oligomerization (compound NSC348884) and the abnormal traffic of NPM1 (avrainvillamide, XPO1 inhibitors) as well as at inducing selective NPM1-mutant protein degradation (ATRA/ATO, deguelin, (-)-epigallocatechin-3-gallate, imidazoquinoxaline derivatives) and at targeting the integrity of nucleolar structure (actinomycin D). We also discuss the current therapeutic results obtained in *NPM1*-mutated AML with the BCL-2 inhibitor venetoclax and the preliminary clinical results using menin inhibitors targeting HOX/MEIS1 expression. Finally, we review various immunotherapeutic approaches in *NPM1*-mutated AML, including immune check-point inhibitors, CAR and TCR T-cell-based therapies against neoantigens created by the *NPM1* mutations.

## Introduction

Nucleophosmin 1 (NPM1) is a ubiquitous nucleus-cytoplasmic shuttling protein [1] predominantly resident in the nucleolus whose functions are pivotal for many cellular processes including histone assembly, centrosome duplication, ribosome biogenesis and export, maintenance of genomic stability and response to nucleolar stress [2].

Mutations of *NPM1* gene are the most frequent genetic lesion in acute myeloid leukemia (AML), being detectable in about one-third of adult AML and 50–60% of AML with normal karyotype [3, 4]. These mutations are a driver genetic lesion and AML defining event that occurs in the context of clonal hematopoiesis, frequently promoted by genes such as *DNMT3A* and *TET2* [4]. Distinctive features of *NPM1*-mutated AML include the mutual exclusion with recurrent cytogenetic abnormalities, the association with specific gene expression [5] and microRNA [6] profiles and the high stability of *NPM1* mutations at relapse [7]. Another characteristic of *NPM1*-mutated AML is the aberrant cytoplasmic dislocation of the NPM1 mutant and NPM1 wild-type proteins (through heterodimerization) [3, 8, 9]. Moreover, the mutant NPM1 is directly involved in promoting high expression of homeobox (HOX) genes [10] which are necessary for maintaining the undifferentiated state of leukemic cells. Notably, this function is closely dependent on the cytoplasmic localization of the mutant. However, the mechanisms underlying leukemogenesis in *NPM1*-mutated AML still remain largely unknown [4]. According to the 5th edition of the World Health Organization (WHO) of hematolymphoid tumors, *NPM1*-mutated AML can be diagnosed irrespective of the percentage of blasts, based on previous observations that cases classified as MDS or MDS/MPN with *NPM1* mutations quickly progressed to AML [11]. Because of the above unique features, *NPM1*-mutated AML is recognized as a distinct entity, within the category of AML with recurrent genetic abnormalities of the WHO classification [11]. The main characteristics of *NPM1*-mutated AML are summarized in Table 1.

**Table 1:** Clinical, pathological and molecular features of *NPM1*-mutated AML.

Main characteristics of *NPM1*-mutated AML
About 30–35% of adult AML (50–60% of AML with normal karyotype). Female predominance
Markedly hypercellular bone marrow. Rare fibrosis. Frequent myelomonocytic (FAB M4) or monocytic (FAB M5) appearance but other FAB categories (except M7) can be represented
Frequent multilineage involvement, as shown by IHC (cytoplasmic NPM1)
Diagnosis can be done irrespective of the percentage of blast cells^a^
Low/moderate WBC count in the absence of *FLT3*-ITD. Progressively increase in WBC when concomitant *FLT3*-ITD and/or *DNMT3A* mutations are present
Frequent extramedullary involvement, especially skin (easily detectable by IHC)
No/low expression of CD34 in the bulk leukemic population. The rare CD34+ leukemic stem cells harbor the *NPM1* mutation
Excellent response to induction chemotherapy
Relatively good outcome in the absence of *FLT3*-ITD. Prognosis may vary depending upon concomitant mutations
Amenable for MRD monitoring by qRT-PCR for *NPM1* mutant transcripts

“AML with cytoplasmic nucleophosmin” (NPM1c) has been also used as a synonym of NPM1-mutated AML.

*IHC* immunohistochemistry, *WBC* white blood cell count, *MRD* measurable residual disease, *qRT-PCR* quantitative reverse transcription polymerase chain reaction.

^a^According to the 5th edition of WHO classification [11].

The standard therapy of *NPM1*-mutated AML in young adults is based upon induction chemotherapy (±FLT3 inhibitors) and consolidation cycles with high/intermediate dose of cytarabine (ARA-C) ± allogeneic hematopoietic stem cell transplantation (HSCT) in first complete remission (CR), depending on the status of the *FLT3* gene and measurable residual disease-MRD [12, 13]. However, despite the remarkable advances in the treatment of *NPM1*-mutated AML, about 50% of patients still died of progressive disease. Thus, there is a need for new therapeutic opportunities. Whole genomic approaches have unraveled the molecular heterogeneity of AML [14, 15] and given a great input to the development of small molecules aimed to target specific genetic abnormalities [16, 17]. Because of their small-size (<500 Da), these compounds can easily penetrate the cell membrane and exert their activity on intracellular proteins involved in cell signaling mechanisms (e.g., kinases) that promote the tumor growth [18]. In the past 5 years, several small molecules have been approved by the Food and Drug Administration (FDA) for AML treatment, including FLT3, IDH1/2 and BCL-2 inhibitors [19, 20]. Contemporarily, the use of immunotherapy as an additional therapeutic strategy has been explored.

Here, we provide an overview of the current status and future perspectives of targeted therapies in *NPM1*-mutated AML.

## Molecular therapeutic targeting of *NPM1*-mutated AML

### Targeting the structure of NPM1

Small molecules can be potentially designed to bind to any portion of a given protein to modulate its function [21]. The multi-domain conformation of NPM1 makes it an appealing target. In fact, NPM1 consists of three domains: the amino-terminal core region (N-term) similar to the members of the nucleophosmin family; the central region involved in the histone binding and the carbossi-terminal region (C-term) unique for NPM1 protein, that is important for its nucleolar localization [2, 22].

The N-term domain contains two nuclear export signals (NES) that promote the shuttling of NPM1 from the nucleus to the cytoplasm through the interaction with the nuclear exporter XPO1 (CRM1, Exportin-1) [9]. The N-term region has also chaperone activity which is crucial for ribosome maturation and oligomerization of NPM1 with other partners and itself [23]. The three-dimensional structure of the N-term consists of eight antiparallel β-strands forming a β-barrel with jelly-roll topology [24]. NPM1 monomers are unfold and intrinsically unstable [23]. However, they are stabilized through formation of a crown-shaped pentamer, with two pentamers interacting in a head-to-head fashion to form a decamer [25]. Association into pentamers is strictly regulated and it is critical for NPM1 functions.

**Fig. 1:**
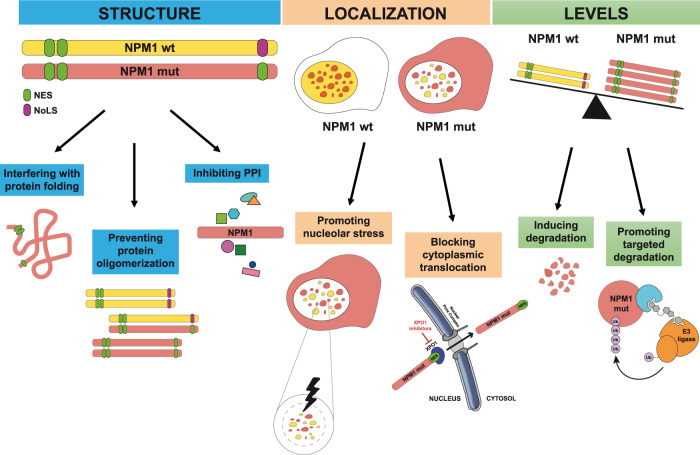
Targeting the structure, localization and levels of the wild-type and mutant NPM1 proteins. Mechanisms of targeting include: interference with protein folding, prevention of NPM1 oligomerization, inhibition of protein–protein interactions (PPIs), promotion of nucleolar stress, block of nuclear export, and induction of protein degradation.

Therefore, disruption of NPM1 oligomers may promote unfolding of the protein and alter NPM1 structure and functions. Targeting NPM1 oligomerization can be achieved using NSC348884, a water-insoluble compound that interacts with the high hydrophobic NPM1 dimerization surface [26]. NSC348884 was more effective in disrupting NPM1 oligomerization and inducing apoptosis in *NPM1*-mutated OCI-AML3 cells than in *NPM1* wild-type HL-60 and OCI-AML2 cells [27] (Fig. 1). This is somewhat surprising because *NPM1* mutations target the C-terminal domain whilst NSC348884 targets the N-terminal domain.

The N-term region is critical for the interaction of NPM1 with its partners through positively charged surface. These molecular interactions are critical to build-up the structure of the nucleolus [28, 29]. In fact, the nucleolus is the result a “liquid-liquid” phase separation leading to the segregation of NPM1 and other components of the nucleolus from the surrounding nucleoplasm [30, 31], similarly to how oil and water separate from each other when mixed. This process is mediated by the interaction of the native pentameric NPM1 molecule with proteins containing R-motifs, i.e., multivalent arginine-rich linear motifs (sharing features with nucleolar localization signals) and nascent ribosomal RNA (rRNA) [29, 32]. Distruption of NPM1 oligomers can interfere with this process.

Many NPM1 interactors that are thought to be involved in leukemogenesis have been claimed to be dislocated in the cytoplasm by the NPM1 mutant [9], although this event has been never conclusively demonstrated in primary *NPM1*-mutated AML cells [4]. Thus, small molecules targeting NPM1 protein–protein interactions could also help preventing downstream effects on important molecules potentially implicated in AML pathogenesis.

The NPM1 C-terminus consists of a three-helix bundle [33] stabilized by strictly conserved aromatic residues (Phe268, Phe276, Trp288, Trp290) [25], and contains the nucleolar localization signal (NoLS) [25]. This aromatic-rich NoLS seems to be rather specific for NPM1. Mutations of tryptophans 288 and 290 (or 290 only) cause unfolding of the three-helix bundle and loss of NPM1 NoLS [9]. This event together with the insertion of a new NES motif, are responsible for the aberrant delocalization of NPM1 in the cytoplasm of AML cells carrying *NPM1* mutations [8, 9].

The C-term of NPM1 mutated protein is unfolded and therefore difficult to target [24, 25]. The only small molecule capable of directly binding this region is avrainvillamide. This natural alkaloid was found to form tight complexes with the NPM1 protein by S-alkylation of cysteine residues [34, 35] and to induce partial nuclear relocalization of mutant NPM1 in OCI-AML3 cells and primary AML cells. This effect was secondary to the ability of avrainvillamide to alkylate specifically Cys275 (in helix H2 of the three-helix bundle at C-terminus) of certain NPM1 mutants [34] and to inhibit the nuclear export of XPO1 cargo proteins, including NPM1 mutants [34]. Thus, avrainvillamide can relocate cytoplasmic NPM1, acting as a substitute for NoLS [34] rather than inducing refolding of the C-terminus three-helix structure of the mutant [34] (Fig. 1). Avrainvillamide was more active against *NPM1*-mutated than wild-type AML cells [36], probably due to the unfolded structure of the C-terminus three-helix bundle, and caused proteasomal degradation of NPM1 mutant and differentiation of OCI-AML3 cells [36]. Moreover, it demonstrated strong anti-proliferative activity against a PDX model of *NPM1*-mutated AML [36].

### Targeting the nucleolus of *NPM1*-mutated cells

NPM1 acts as a nucleolar stress sensor [37]. The nucleolus of *NPM1*-mutated AML cells may be particularly vulnerable to stress because it is NPM1 depleted due to both haploinsufficiency and cytoplasmic delocalization [38]. Therefore, inducing nucleolar stress represents a therapeutic option in *NPM1*-mutated AML [39] (Fig. 1). This prompted us to evaluate actinomycin D which, at low dosage, is a selective inhibitor of RNA polymerase I and ribosome biogenesis. This drug induced nucleolar stress in *NPM1*-mutated cells [40, 41] and CR in relapsed/refractory *NPM1*-mutated AML [38, 40]. Other drugs causing nucleolar stress through inhibition of ribosome biogenesis [42] should be investigated in *NPM1*-mutated AML.

However, the mechanism of action of actinomycin D in *NPM1*-mutated AML may be even more complex. In fact, NPM1 mutants can impede the formation of acute promyelocytic leukemia (APL) nuclear bodies (NBs) [43], which in turn are regulators of mitochondria fitness and key senescence effectors [44]. Thus, *NPM1*-mutated AML cells are characterized by defective mitochondrial function [44]. Actinomycin D acts on NPM1 mutant-primed mitochondria by releasing mitochondrial DNA, activating cyclic GMP-AMP synthase signaling, producing reactive-oxygen species (ROS) that restores NB formation to drive TP53 activation and senescence of *NPM1*-mutated AML cells. Interestingly, actinomycin D appears to potentiate the activity of venetoclax on mitochondrial function and apoptosis [44].

### Targeting NPM1 protein levels

Targeting the levels of mutant NPM1 represents another therapeutic opportunity (Fig. 1). Combining arsenic trioxide (ATO) with all-trans retinoic acid (ATRA) resulted into proteasome-dependent degradation of NPM1 oncoprotein and death in AML cell lines and primary AML cells carrying *NPM1* mutations [43, 45]. Treatment of the *NPM1*-mutated AML cell lines OCI-AML3 with deguelin, a rotenoid isolated from several plant species [46, 47], and IMS-M2 with (-)-epigallocatechin-3-gallate (ECGT) [48], a major catechin found in green tea, were effective in reducing the NPM1 mutant but not the wild-type protein and in inducing apoptosis. Moreover, the imidazoquinoxaline derivative EAPB0503 induced a selective proteasome-mediated degradation of NPM1 mutant protein through EAPB0503-mediated SUMOylation and ubiquitylation [49]. This event was followed by restoration of NPM1 wild-type protein in the nucleolus [50], apoptosis (through selective downregulation of HDM2 and activation of *p*53 [49]) and reduction of leukemia burden in *NPM1*-mutated AML xenografts [49, 50]. Moreover, introducing *NPM1*-mutation into cells normally bearing wild-type NPM1 sensitized them to EAPB0503, leading to their growth arrest [50].

Mutant NPM1 can be also targeted through proteolysis targeting chimera (PROTAC) which allows ubiquitination and proteasome-mediated degradation of the target protein [51] (Fig. 1). PROTAC promoted degradation of fused oncoproteins in MLL leukemia subtypes [52] and its use could be extended to *NPM1*-mutated AML. In this regard, selective degradation of mutant NPM1 through degron-tag, invariably induced differentiation, and growth arrest of *NPM1*-mutated cell lines [10], confirming the validity of such approach.

### Targeting NPM1 localization

The multilayered nucleolar structure (the fibrillar core, surrounded by the dense fibrillar component and the granular component) is dictated by the “liquid-liquid” phase separation involving nucleolar proteins, primarily native NPM1 and fibrillarin that segregate in the granular component and dense fibrillar component, respectively [30]. NPM1 is predominantly localized in the nucleolus, and shuttles to the cytoplasm as a consequence of the interaction of the two N-terminal NES with XPO1. XPO1 recognizes and binds also other NES-containing molecules including tumor-suppressor proteins [53, 54], promoting their export from the nucleus to the cytoplasm.

Altered nuclear-cytoplasmic shuttling is a peculiar characteristic of *NPM1*-mutated AML [9]. Thus, targeting nuclear export is an appealing strategy in this pathological condition (Fig. 1). The natural compound leptomycin B shows strong XPO1 inhibitory activity in vitro [55] but its irreversible binding to XPO1 is associated with severe toxicity [56]. More recently, inhibitors that bind reversibly to XPO1 have become available. These drugs, collectively known as selective inhibitors of nuclear export (SINEs), include KPT-185, KPT-249, KPT-251, KPT-276, KPT-330, and KPT-335 [57] (Table 2). Among them, KPT-330 (selinexor) is the most well-known SINE compound that has been also tested in AML. [58] However, selinexor was scarcely active towards *NPM1*-mutated AML patients in early-phase clinical trials [59–61]. This is likely due to the once or twice/week administration schedule of selinexor (due to its toxicity profile) which is not sufficient to stably inhibit the interaction between mutated NPM1 and XPO1 (Pianigiani et al. BioRxiv 2021). An attractive alternative to selinexor is the second-generation SINE compound KPT-8602 (eltanexor) that crosses at lower extent the blood-brain barrier resulting in better tolerability. More importantly, the prolonged and frequent dosing schedule of eltanexor (i.e., 5 days/week) led to a greater anti-leukemic efficacy in preclinical animal models of hematological malignancies [62, 63]. This 5 days/week eltanexor schedule resulted into more robust anti-leukemic activity than selinexor alone in models of *NPM1*-mutated AML cells in vitro and in vivo (unpublished data). Interestingly, eltanexor synergizes with a BCL-2 inhibitor by increasing apoptosis in primary AML cells [64].

**Table 2:** Summary of in vitro and in vivo data of given compounds and related clinical trials.

Drug	Target	Mechanism of action	In vivo and in vivo efficacy^a^	Clinical trial (ClinicalTrials.gov Identifier)	Phase; Patients; Recruitment Status	Combination therapies	Clinical outcome in *NPM1*-mut AML pts (CR/CRi)
Leptomycin B (LMB)	XPO1 inhibitor natural compound (irreversible)	NPM1 export inhibition	OCI-AML2, OCI-AML3, HL-60, KG1, MV4-11; RPMI-8226 and NCI-H929 (MM cell lines).	/	/	/	/
CBS9106	XPO1 degradation (reversible)	NPM1 export inhibition	MM.1R, MM.1S, RPMI-8226, and ARH-77 (MM cell lines). RPMI-8226 xenograft mice.	/	/	/	/
KPT-185	XPO1 inhibitors (reversible) SINE, 1st generation	NPM1 export inhibition	HL-60, Kasumi-1, KG1a, MOLM13, MV4-11, OCI-AML3, and THP-1 cell lines; NCI-H929 and RPMI-8226 (MM cell lines). AML primary patient samples with *NPM1* and *FLT3* -ITD mutations. Primary CLL cells.	/	/	/	/
KPT-249			HL-60; APL cell lines; NCI-H929 and RPMI-8226 (MM cell lines).	/	/	/	/
KPT-251			Primary CLL cells; MV4-11 xenograft mice.	/	/	/	/
KPT-276			HL-60, MV4-11; NCI-H929 and RPMI-8226 (MM cell lines). MV4-11 xenograft mice.	/	/	/	/
KPT-330 (Selinexor)			HL-60, K562, KG-1, IMS-M2, MOLM13, MOLM16, MOLT-4, MV4-11, NB4, OCI-AML3, OCI-AML5, THP-1 and U937 cell lines; NCI-H929 and RPMI-8226 (MM cell lines). MV4-11 xenograft mice and others AML-derived xenografts (including CN and *FLT3* -ITD).	NCT02093403	Phase 1; 25 patients; Enrollment completed	Decitabine	Evaluating efficacy
				NCT02249091	Phase 2; 42 patients; Enrollment completed	Ara-C and Idarubicin	Evaluating efficacy
				NCT02088541	Phase 2; 317 patients; Enrollment completed	Hydroxyurea and Ara-C	Evaluating efficacy
				NCT01607892	Phase 1; 286 patients (95 AML patients); Enrollment completed	/	Evaluating efficacy
				NCT03955783	Phase 1b; 78 patients; Recruiting	Venetoclax	Evaluating efficacy
KPT-335			Jurkat (T-cell leukemia); OCI-Ly3 and OCI-Ly10 (B-cell lymphoma) cell lines; CLBL1 (canine lymphoma) cell line; primary DLBCL cells.	/	/	/	Evaluating efficacy
KPT-8602 (eltanexor)	XPO1 inhibitors (reversible) SINE, 2nd generation	NPM1 export inhibition	K562, Kasumi-1, KG1, MOLM13, MOLM16, Mono-Mac-1, MV4-11, NB4, OCI-AML2, OCI-AML3, SKM1, and U937. MV4-11 xenograft mice.	NCT02649790	Phase 1/2; 119 patients;Recruiting	ASTX727 and Dexamethasone	Evaluating efficacy
VTX (Venetoclax)	BCL-2 inhibitor	Apoptosis	GDM1, HL-60, KG1, K562, ME1, ML2, MOLM13, MOLM16, Mono-Mac-6, MV4-11, OCI-AML2, OCI-AML3, OCI-M1, NB4, NOMO1, THP-1 and U937. AML primary patient samples with *NPM1*, *FLT3*-ITD mutations. MV4-11 xenograft mice and others AML-PDX models harboring different mutations including *NPM1, DNMT3A, FLT3, IDH1*, *PML-RARα*, *CEBPA*.	NCT04867928	Phase 2; 35 patients; Recruiting	Azacitidine	Evaluating efficacy
				NCT02203773	Phase 1b; 212 patient Active, not recruiting	Azacitidine or Decitabine	91.5% (*N* = 23, 21/23)
				NCT02993523	Phase 3; 400 patients; Active, not recruiting	Azacitidine	66.7% (*N* = 27, 18/27)
				NCT02287233	Phase 1/2; 94 patients; Enrollment completed	LDAC	89% (*N* = 9, 8/9)
				NCT03069352	Phase 3; 211 patients; Active, not recruiting	LDAC	78% (*N* = 18, 14/18)
				ACTRN12616000445471^b^	Phase 1b; 48 patients; Recruiting	5 + 2 (cytarabine + idarubicin)	80% (*N* = 10, 8/10)
				NCT03214562	Phase 1b/2; 116 patients; Recruiting	FLAG + IDA	100% (*N* = 8, 8/8)
MI-2-2	MLL-Menin protein–protein interaction inhibition	HOX genes and MEIS1 downregulation	HL-60, KOPN-8, ML-2, MOLM13, MV4-11 and OCI-AML3 cell lines; MLL-AF9, MLL-AF6 and MLL-AF1p. OCI-AML3 xenograft mice.	/	/	/	/
MI-503			MV4-11 and OCI-AML3 cell lines. MLL-AF9 BMCs from patients. MOLM13, MV4-11 and OCI-AML3 xenograft mice.	/	/	/	/
MI-3454			K562, KOPN-8, MOLM13, MV4-11, SET2 and U937. RS4-11 and SEM (B-cell leukemia) cell lines. AML primary patient samples with *NPM1* mutations or *MLL1* translocations. MOLM13 and MV4-11 xenograft mice.	/	/	/	/
KO-539			MOLM13, MV4-11, OCI-AML3 cell lines. AML primary patient samples with *NPM1, KMT2A* and *FLT3-TKD* mutations. PDX models with *MLL-FP* or *NPM1* mutations.	NCT04067336 (KOMET-1)	Phase 1/2; 60 patients; Recruiting	FLT3 inhibitors	Evaluating efficacy
VTP-50469	MLL-Menin protein–protein interaction inhibition	HOX genes and MEIS1 downregulation	HL-60, K562, ML2, MOLM13, MV4-11, NOMO1, OCI-AML3, THP1 cell lines. RS4-11, KOPN-8 and HB11-19 (B-cell leukemia) cell lines. Mouse MOZ-TIF2 cells and MLL-AF9 cells. MV4-11 xenograft mice and others AML-PDX models harboring different mutations including *NPM1, FLT3, DNMT3A* and *IDH1*.	/	/	/	/
SNDX-5613			MOLM13, MV4-11 and OCI-AML3. MOLM13 xenograft mice and others AML-PDX models harboring different mutations including *NPM1, DNMT3A, FLT3, IDH1, WT1, KMT2C*.	NCT04065399 (AUGMENT-101)	Phase 1/2; 186 patients; Recruiting	/	30% (*N* = 10, 3/10)
JNJ-75276617			AML cell lines (not specified). AML patient samples with *NPM1* and *KMT2Ar* mutations.	NCT04811560	Phase 1; 110 patients; Recruiting	FLT3 inhibitors	Evaluating efficacy
DS-1594b	Menin inhibitor	HOX genes and MEIS1 downregulation	AML and ALL cells with *MLLr* (not specified).	NCT04752163	Phase 1/2; 122 patients; Recruiting	Azacitidine, Venetoclax, or mini-HCVD	Evaluating efficacy
BMF-219	Menin inhibitor (irreversible)	HOX genes and MEIS1 downregulation	MOLM13 cell line. CLL and MM cell lines (not specified). AML patient samples with *NPM1* and *MLLr* mutations.	NCT05153330 (COVALENT-101)	Phase 1; 100 patients; Recruiting	/	Evaluating efficacy

*CR* complete response, *CRi* complete response with incomplete count recovery, *CN* cytogenetically normal, *SINE* selective inhibitor of nuclear export, *MM* Multiple myeloma, *DLBCL* diffuse large B-cell lymphoma, *HMAs* hypomethylating agents, *LDAC* Low-Dose Cytarabine, *mini-HCVD*  mini–hyper fractionated cyclophosphamide, vincristine and dexamethasone, *FLAG-IDA* fludarabine, cytarabine, granulocyte colony-stimulating factor (G-CSF) and idarubicin.

^a^Data from the most important studies in AML cells are reported, unless otherwise indicated.

^b^Australian Clinical Trials.

## Targeting of other NPM1 pathways

### Targeting apoptosis pathway

**Fig. 2:**
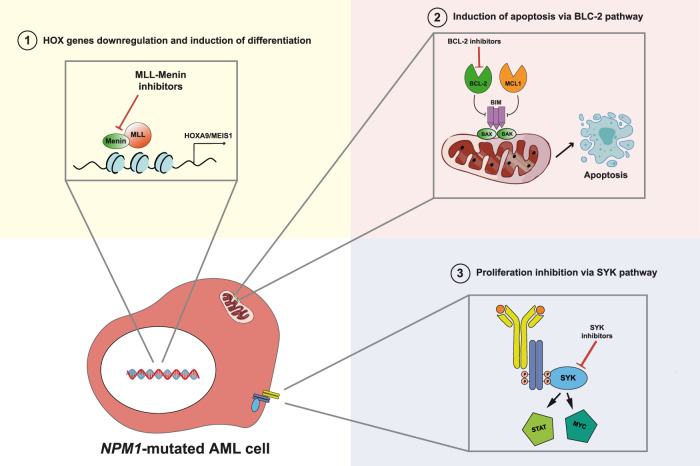
Other approaches to target *NPM1*-mutated AML. *NPM1*-mutated AML can be targeted with selective inhibitors of menin to downregulate HOX/MEIS, with BCL-2 (venetoclax) to induce apoptosis and with inhibitors of the SYK pathway (entospletinib).

The BCL-2 family of proteins play a key role in the intrinsic mitochondrial apoptotic response and BCL-2 is a key survival factor in AML. The anti-apoptotic proteins BCL-2 and MCL1 inhibit apoptosis by sequestering the pro-apoptotic protein BIM, which is required for activation of BAX/BAK and the subsequent induction of mitochondrial outer membrane permeabilization. Venetoclax, a selective BCL-2 inhibitor, when combined with hypomethylating agents (e.g., 5-azacytidine) or low-dose cytarabine (LDAC), shows anti-leukemic activity in 60-70% of AML patients [65]. By downregulating MCL1 and inducing the expression of the pro-death proteins NOXA and PUMA, azacytidine inhibits synergistically the pro-survival proteins MCL1 and BCL-XL, increasing the dependence of leukemia cells on BCL-2. Moreover, venetoclax (with 5-azacytidine) induces leukemic stem cells toxicity by decreasing amino acid uptake, which is essential for oxidative phosphorylation and survival [66, 67]. Thus, venetoclax + 5-azacytidine (or decitabine) and venetoclax + LDAC have become the standard treatment for newly diagnosed older or unfit AML patients [12, 65]. In keeping with preclinical studies [68], *NPM1*-mutated AML patients appear to be particularly sensitive to venetoclax (Fig. 2). Whether this efficacy is related to the high expression of *HOX* genes [5], that in turn are linked to BCL-2 inhibitor sensitivity and responsiveness [69], remains to be defined. NPM1 mutant-primed defect in mitochondrial function may also be responsible for the higher sensitivity to venetoclax [44].

*NPM1*-mutated AML particularly benefits from venetoclax-based regimens, both at first diagnosis [70, 71] (independently by the *FLT3* status [72]) and in the relapsed/refractory setting [73–75] (Table 2). In the phase 3 clinical trial (NCT02993523) of venetoclax plus azacytidine, 66.7% of *NPM1*-mutated AML patients achieved CR + CRi [65], a majority of them being negative for measurable residual disease (MRD). Similar good results have been reported in real-world [76]. One-year OS for elderly patients with *NPM1*-mutated AML exceeded 80%, with an estimated 2-year OS of 70% [70]. Usually, the response was reached with 1–2 cycles and a good safety profile [65]. In trials NCT02287233 and NCT03069352, venetoclax plus LDAC resulted into CR + CRi of 89% and 78%, respectively, in *NPM1*-mutated AML patients [77, 78]. CR and CRi of 80% and 100%, were also achieved in *NPM1*-mutated AML patients, when venetoclax was combined with 5 + 2 (cytarabine + idarubicin) or FLAG + IDA (fludarabine, cytarabine, granulocyte colony-stimulating factor, and idarubicin) (ACTRN12616000445471, NCT03214562) [79, 80].

**Fig. 3:**
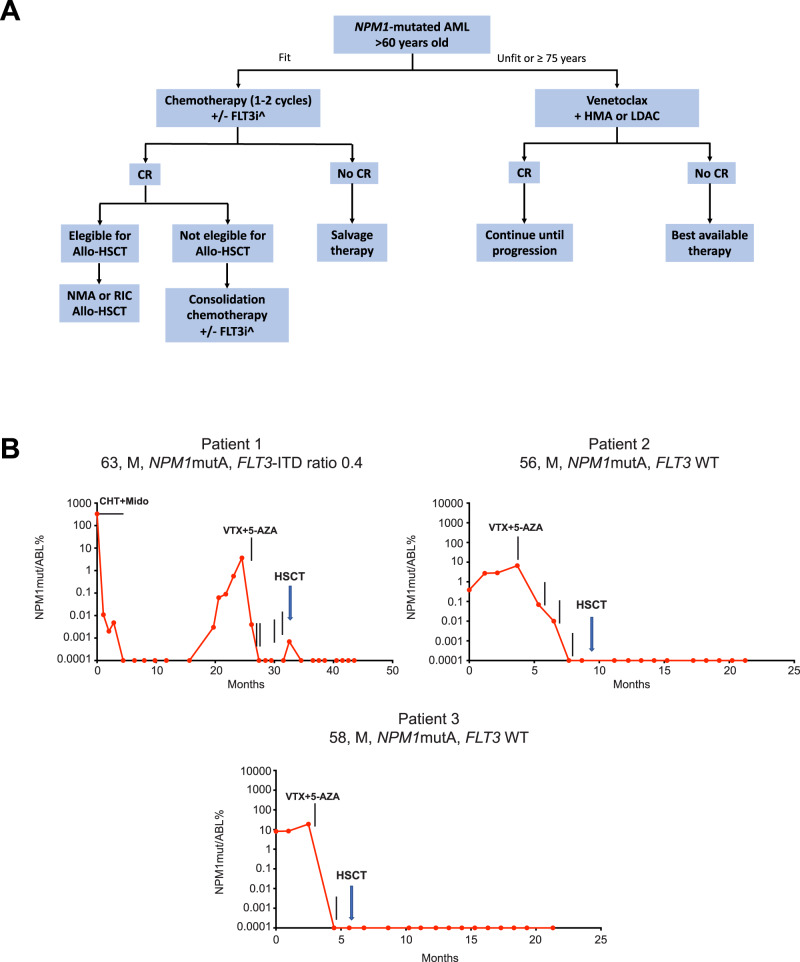
Venetoclax in *NPM1*-mutated AML. **A** Algorithm for the treatment of *NPM1*-mutated AML patients older than 60 years. ^Based on the presence or absence of *FLT3* mutations. CR complete remission, FLT3i FLT3 inhibitors, HMA hypomethylating agents, LDAC low-dose cytarabine, allo-HSCT allogeneic hematopoietic stem cell transplantation. **B** Examples of preemptive therapy with venetoclax as bridging to allo-HSCT. All three patients achieved molecular CR (negativity for *NPM1* mutant transcripts) before allo-HSCT. VTX venetoclax, 5-AZA 5-azacytidine, CHT chemotherapy.

CR rates in refractory/relapsed *NPM1*-mutated AML are lower, ranging between 46% and 66.8%. In general, venetoclax plus 5-azacitidine was associated with better results than venetoclax plus decitabine or LDAC. Therapy was usually administered continuously until progression. However, about half of AML patients carrying *NPM1* and/or *IDH2* mutations and/or achieving a molecular CR after at least 12 months of a venetoclax-based regimen, experienced a long treatment-free remission, after therapy cessation [81]. Venetoclax-based regimens have been also used pre-emptively to treat 12 *NPM1*-mutated AML patients with persistent or relapsed/progressed MRD [82]. All five patients with persistent MRD and 6/7 patients with relapsed/progressed MRD, achieved durable molecular CR, after 1–2 cycles of venetoclax, with <17% grade >3 non-hematological toxicities [82]. Thus, venetoclax-based therapies have the potential to be used for *NPM1*-mutated MRD-positive patients, as bridge to allotransplant (Fig. 3A, B).

Unfortunately, most patients treated with these regimens develop resistance over time and eventually relapse. To circumvent this problem, new combinations are currently tested, including venetoclax plus XPO1 [64] or menin [83, 84] inhibitors. In mouse models of *NPM1*-mutated/*FLT3*-ITD AML, venetoclax plus menin inhibitor was superior to the menin inhibitor alone, in eliminating leukemic cells (including leukemia stem/progenitor cells), decreasing Bcl-2 and Bcl-xL levels [83], and significantly prolonging mice OS [83]. ATO can potentiate the activity of venetoclax by attenuating Mcl-1 upregulation induced by venetoclax in AML cells. ATO plus venetoclax synergistically induced apoptosis in OCI-AML3 cells in vitro and were active in two R/R *NPM1*-mutated AML patients [85].

### Targeting the MLL-menin complex

The *Menin* (*MEN1*) gene is located on chromosome 11q13 and encodes for a protein which regulates tissue-specific gene expression [86]. In human, germline *MEN1* mutations cause the multiple endocrine neoplasia type-1 syndrome [87]. In mice, knock-out of the *Men1* gene leads to reduced expression of *Hoxc6* and *Hoxc8* genes during embryogenesis [88]. Menin is a co-factor of the histone-lysine-N-methyltransferase 2A (KMT2A) that induces the trimethylation of lysine 4 on histone 3, a histone mark that correlates with active transcription of HOX genes and their co-factor MEIS1 (HOX/MEIS). HOXA and HOXB gene clusters are highly expressed in normal adult hematopoietic stem/progenitor cells but are physiologically silenced in mature blood cells, suggesting their role in self-renewal [89]. High expression of HOXA and HOXB cluster genes and their co-factor *MEIS1* contributes to the gene signature typical of *NPM1*-mutated AML [4, 5]. Interestingly, the HOXA and HOXB expression levels in *NPM1*-mutated AML overlap with those of normal hematopoietic stem/progenitor cells, pointing to common mechanisms regulating HOX expression and to persistent expression rather than upregulation of HOX genes in *NPM1*-mutated AML [90].

More recently, we proved that HOX expression is directly dependent upon the aberrant cytoplasmic dislocation of NPM1 mutants [10]. Indeed, nuclear relocalization or targeted degradation of cytoplasmic mutated NPM1 resulted into the rapid loss of HOX expression, followed by differentiation and growth arrest [10]. However, the link between cellular localization of NPM1 mutants and HOX expression remains unclear. A possible model implies that NPM1 directly binds nucleolar putative factors that repress HOX genes required for proper differentiation and displaces them from the nucleus to the cytoplasm [4]. This would be in keeping with the finding that nuclear relocalization of NPM1 mutants from the cytoplasm to the nucleus restore the normal activity of these factors, leading to downregulation of HOX genes [4]. Alternatively, recruitment of NPM1 mutants to HOX loci through their interaction with chromatin-bound XPO1 [91] could be responsible for HOX expression maintenance, cytoplasmic delocalization of NPM1 representing a mere epiphenomenon. We previously suggested that NPM1 mutants may induce leukemia by acting both at the chromatin level and by delocalizing NPM1-interacting partners in the cytoplasm [4].

Dependence of *NPM1*-mutated AML cells on epigenetic machinery for HOX regulation [92] provides the rationale for using inhibitors of KMT2A-menin protein interaction [93, 94] (Fig. 2; Table 2). The VTP-50469 inhibitor showed strong in vitro and in vivo anti-leukemic activity on *NPM1*-mutated AML cells, causing a dose-dependent reduction in cell proliferation, a significant downregulation of HOXA/B clusters and *MEIS1* gene expression, a marked differentiation of leukemic cells, a reduction of AML engraftment and a prolonged survival in mice PDX models [92, 95, 96]. This occurred especially through rapid repression of important co-factors of HOX genes (*MEIS1* and *PBX3*), the effect on expression of HOXA and HOXB genes being not relevant [97]. The MI-3454 inhibitor was very effective in inhibiting cell proliferation and differentiation of *NPM1*-mutated AML cells, independently from the coexistence of other mutations in patients’ samples, and in reducing blast infiltration of organs and expression of *MEIS1* and *FLT3* in PDX *NPM1*-mutated mouse models [95].

Menin inhibitors were reported to prevent the transformation of *Npm1*-mutated mouse hematopoietic progenitors into leukemic cells, implying that they could also be effective in *Npm1*-mutated preleukemia [96]. However, we believe that this concept is difficult to translate into clinic because *NPM1* mutations in patients do not associate with a preleukemic state [4]. Moreover, myelodysplasia with *NPM1* mutations is very rare and cases with these characteristics usually represent already early-stage AML [12]. Thus, we expect that the value of menin inhibitors in clinic will be mainly limited to the therapy of frank *NPM1*-mutated AML.

Menin inhibitors was also combined with inhibitors of *FLT3* (mutated in about 40% of *NPM1*-mutated AML [3]), demonstrating a synergistic effect that resulted in stronger cell growth inhibition, apoptosis and differentiation of AML blast cells [98] and induction of long-lasting CR in PDX mice models of *NPM1*-mutated/*FLT3*-ITD AML [99]. Menin inhibitors have been also combined with XPO1 inhibitors. The rationale for this association is that nuclear relocation of the NPM1 mutant by the XPO1 inhibitors is associated with downregulation of HOX genes [10]. Thus, the two compounds could exert a cumulative effect on downregulation of HOX genes through different mechanisms. The utility of combining menin inhibitors with venetoclax has been already mentioned [83]. All these associations could help preventing the rapid development of resistance to targeted monotherapies.

Menin inhibitors, including KO-539, SNDX-5613, JNJ-75276617 and DS-1594b, are currently evaluated in clinical trials [100, 101]. KO-539 induced CR in 2/6 patients with R/R AML who were evaluable for efficacy analysis. One of them had an *NPM1*-mutated AML co-mutated for *DNMT3A* and *KMT2D* who received KO-539 at 200 mg/die, as the eight line of therapy, achieving an MRD-negative CR [101]. This trial continues to enroll patients with *NPM1*-mutated and *KTM2A*-rearranged AML on the doses of 200 and 600 mg (NCT04067336). SNDX-5613 (an analog of VTP-50469) was very active in PDX mouse models of *NPM1*-mutated AML, including animals remaining in CR 1 year after cessation of therapy [96, 102]. The safety and efficacy of SNDX-5613 in adult patients with R/R *NPM1*-mutated or *KTM2A*-rearranged AML is being evaluated in the AUGMENT-101 phase I/II trial (NCT04065399). Preliminary results of this study were released by Syndax in April 2021. By March 2021, the trial had enrolled in the phase 1 cohort, 43 patients (median age 54 years) who had received a median of 3 previous lines of treatment. The most common side effects (>5%) included QT prolongation (14%), differentiation syndrome (5%) and anemia (5%). The overall response rate in the 7 patients with *NPM1*-mutated AML was 29% (2/7). In keeping with the proposed mechanism of menin inhibitors, RNA-Seq analysis of the bone marrow samples from responding patients exhibited downregulation of *MEIS* and *HOXA9* genes and upregulation of the differentiation antigens CD11b, CD14 and CD13. The phase 2 part of the trial is ongoing. JNJ-75276617 is a potent inhibitor of the binding between menin and KTM2A. Its safety and activity are being tested in NCT04811560 that enrolls AML patients harboring *NPM1* mutations or *KMT2A* rearrangements. However, no data have been released so far. The safety and efficacy of DS-1594b menin inhibitor will be evaluated as single drug or in combination with azacytidine and venetoclax regimens in a phase 1/2 clinical trial (NCT04752163). To maximize the depth and durability of clinical response, the Biomea Fusion, Inc. has recently developed BMF-219, an orally bioavailable, potent and selective irreversible covalent menin inhibitor. Clinical trial with this compound is ongoing (NCT05153330).

### Targeting SYK signaling

In a phase 1b/2 trial (NCT02343939), entospletinib, a selective oral inhibitor of the spleen tyrosine kinase (SYK) which is constitutively activated in AML promoting survival and proliferation [103], when combined with chemotherapy, was more active in patients with *HOXA9/MEIS1* signature (as in *NPM*1-mutated AML) than in the whole patient population [104] (Fig. 2). These results suggest that the increased expression and activity of SYK protein is strictly dependent upon the deregulation of *HOXA* and *MEIS* genes [105]. Based on this evidence, FDA approved a phase 3 trial to assess the efficacy and safety of entospletinib in combination with chemotherapy in adult patients with newly diagnosed *NPM1*-mutated AML (NCT05020665).

## Immunotherapy of *NPM1*-mutated AML

Ideally, any target antigen for AML immunotherapy should be expressed at high levels in the whole leukemic population, including leukemic stem cells, and to be absent or low expressed in normal hematopoietic cells and other tissues. Leukemia-associated antigens (e.g., CD33 and CD123) are usually strongly expressed in AML cells (especially in *NPM1*-mutated AML cells [106–108]) but can also be detected in normal hematopoietic stem cells and in extramedullary tissues (e.g., CD123 in endothelial cells). This limits their use as target antigens for immunotherapy because of potential off-target effects.

Conversely, leukemia-specific antigens deriving from altered proteins encoded by leukemogenic mutations (e.g., *NPM1*), are specifically expressed in malignant clones and therefore represent ideal targets. In particular, the *NPM1* mutant neoantigen can be considered an ideal AML target for a number of reasons. First, *NPM1* mutations are common driver, gate-keeper events [109], very stable at relapse [4], specific for AML and absent in normal tissues [110]. Second, the *NPM1* mutated proteins are detectable in chemoresistant leukemic stem cells [108], making them possibly vulnerable to immune surveillance and eradication. Third, although >50 *NPM1* mutations have been identified, the 4 bp frameshift insertion occurring in *NPM1* mutant A is responsible for almost 80-85% of all mutations and more rare *NPM1* mutations lead to the same amino acidic changes at NPM1 C-terminus. Fourth, the newly acquired amino acid C-terminus sequence of NPM1 mutant proteins is highly immunogenic in animals, eliciting specific antibodies. Fifth, the aberrant localization of the NPM1 mutant proteins in the cytoplasm of leukemic cells may favor their processing by the Human Leukocyte Antigen (HLA) MHC class I degradation pathway leading to HLA presentation and anti-cancer immune response. Indeed, using in silico analysis, we predicted that several peptides could bind to specific HLA class I molecules [111]. Sixth, specific autologous cytotoxic T-cell responses against NPM1 mutant peptides could be detected in *NPM1*-mutated AML patients [112–115]. These immune responses associated with molecular CR [116] and may explain the relatively favorable outcome of *NPM1*-mutated AML [117]. interestingly, *NPM1*-mutated AML sensitivity to T-cell immunity has been observed not only in the autologous but also in allogeneic setting. Although *NPM1*-mutated AML patients without *FLT3-*ITD has a good prognosis, those who underwent allogeneic HSCT showed a particularly long-term disease control [118], probably due to specific graft-versus leukemia effect. Moreover, polyspecific T-cell anti-leukemic responses, even against NPM1-mutated peptides, have been observed following preemptive donor lymphocyte infusions (DLIs) at molecular relapse after allogeneic HSCT [116]. Finally, the importance of eradicating the *NPM1*-mutated clone to achieve cure of AML is exemplified by the clinical observation of patients with *NPM1*-*DNMT3A* double-mutated AML after cessation of therapy. These cases, when achieve long-term molecular (MRD-negative) remission, are likely to be cured, even though the persistence of detectable copies of the *DNMT3A* mutant (indicating persistent clonal hemopoiesis) may expose them to a low risk of developing a second AML [4].

### Antibodies against CD33 and CD123

**Fig. 4:**
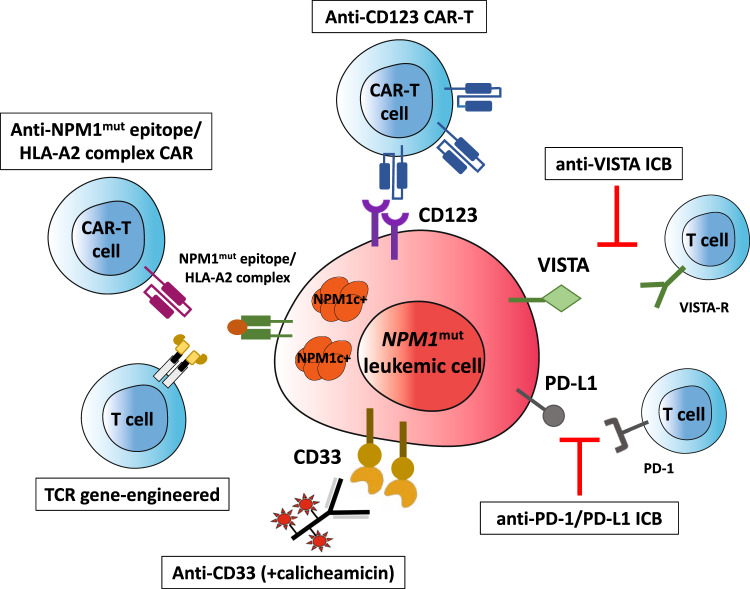
Immunotherapeutic approaches to *NPM1*-mutated AML. *NPM1*-mutated AML can be targeted using antibody-drug conjugates (e.g., gentuzumab ozogamicin, anti-CD33), immune check-point inhibitors, CAR and TCR-based adoptive T-cell therapies directed against NPM1 mutated epitope/HLA complex. CAR chimeric antigen receptor, TCR T-cell receptor.

CD33 is expressed in all stages of myeloid differentiation [119] and it is detectable in most cases of AML, the expression levels being high in *NPM1*-mutated AML [106]. Thus, CD33 is an useful target for immunotherapy with an anti-CD33 monoclonal antibody conjugated with a DNA-damaging calicheamicin derivative (Gemtuzumab Ozogamicin-GO) (Fig. 4). In a metanalysis study [120], adding GO to chemotherapy showed a survival benefit for intermediate-risk cytogenetics and *NPM1*-mutated AML patients because of a reduced relapse risk. Similar results were reported by the ALFA0701 trial that also demonstrated the impact of GO in reducing *NPM1*-mut transcripts level [121]. Higher reduction of *NPM1*-mut transcript levels were also observed in the GO arm of the AMLSG study that translated into a lower cumulative incidence of relapse [122]. However, the AMLSG 09-09 Phase III Study failed to meet the early primary end point (event-free survival) due to higher early mortality in the GO arm [123]. Nevertheless, a significant clinical benefit was observed in females older than 70 years with *NPM1*-mutated/*FLT3* wild-type genotype [123] in terms of both event-free-survival and cumulative incidence of relapse. Collectively, the above studies support the incorporation of GO into the frontline treatment of *NPM1*-mutated AML.

CD123 is highly expressed in *NPM1*-mutated AML both at diagnosis and relapse (Fig. 4), the highest expression being observed in CD34+ CD38− leukemic cells [107]. Moreover, CD123 expression was enhanced by *FLT3* mutations, suggesting that the subset of *NPM1/FLT3* double-mutated AML patients could particularly benefit from anti-CD123 targeted therapies [107]. So far, tagraxofusp (SL401) which is formed by the fusion of IL-3 with diphtheria toxin and the CD123-directed chimeric antigen receptor (CAR) T cells (MB-102) developed by Mustang Bio Inc. are approved or received the Orphan drug designation (MB-102) for the treatment of blastocytic plasmacytoid dendritic cell neoplasm [124] which strongly expresses CD123. Only scarce information is available in CD123-positive R/R AML. Main limitation of these products is myelotoxicity [124].

### Antibodies against PD-1 and PD-L1

AML is a poorly immunogenic and highly immune-suppressive hematological malignancy. High expression of PD-L1 has been found in *NPM1*-mutated AML patients, especially in the leukemic progenitors/stem cell compartment (CD34+ CD38−) [125]. High PD-L1 expression in blasts of AML with *NPM1*-mutated/*FLT3*-ITD genotype predicted inferior survival [41]. Moreover, in a comprehensive immunogenomic analysis of AML, mutations of *NPM1* and *FLT3* preferentially associated with low T-cell cytolytic activity and a reduced expression of HLA-II (and/or related genetic determinants of HLA-II expression, as *CIITA*) [126]. Interestingly, *CIITA* methylation may limit antigen presentation by primary *NPM1*^mut^*IDH1*^mut^ AML blasts through downregulation of MHC-II, thereby inducing immune evasion [126]. Immune evasion in *NPM1*-mutated AML is also contributed by VISTA (V-domain Ig suppressor of T-cell activation) and ULBP1 (NKG2 ligand) immunoregulatory circuitries that are both significantly upregulated in *NPM1*-mutated AML patients [126] (Fig. 4). VISTA-Ig, which shows a partial homology with other B7 family members, is predominantly expressed in hematopoietic cells of myeloid lineage. This circuitry in mainly involved in suppressing proliferation of T cells and blunting the production of T-cell cytokines, making it a potential target for immune check-point blockade combinations [127].

More recently, the anti-PD-1 antibody, nivolumab, was found to increase leukemia-associated antigen-stimulated cytotoxic T cells and cytotoxicity against stem cell-like cells, especially those carrying *NPM1* mutations [128]. These findings provide a rationale for the treatment of *NPM1*-mutated AML, combining anti-PD-1 and anti NPM1-mutation specific immunotherapy (see below). Moreover, targeted immune gene expression and multiplexed digital spatial profiling showed distinct AML immune microenvironments [129]. The *immune-infiltrated* microenvironment that was characterized by severe immune suppression (high expression of *PD-L1*, *CTLA4*, *IDO1* and *BTLA)*, higher dependence from IFN-γ driven adaptive immune responses, high T-cell infiltration and expression of major histocompatibility complex, closely clustered with the adverse-risk genetic AML categories (specifically, *TP53* and *RUNX1* mutated AML) [129]. Conversely, the *NPM1*-mutated cases (with or without *FLT3*-ITD) more frequently showed an *immune-depleted* microenvironment [129]. Finally, *NPM1*-mutated AML harboring concomitant clonal hematopoiesis driven mutations (e.g., *DNMT3A*, *TET2*) showed an enriched tumor inflammation signature score, predicting a clinical benefit from anti-PD-1 treatment [130]. This finding is in keeping with the observation linking in a mouse model a persistent immune stimulation to an accelerated *NPM1*^mut^ myeloproliferative phenotype in vivo [131].

Although the above findings suggest that *NPM1*-mutated AML may be a potential candidate for immune check-point inhibition (Fig. 4), the few studies performed so far with anti-PD-L1 antibodies in AML patients have shown only modest clinical activity. Their impact has been evaluated also in combination with hypomethylating agents [132–134], since they induce the expression of several immune-related genes, including HLA-I and HLA-II, leukemia-associated antigens (e.g., PRAME, WT1) [135], PD-1 and PD-L1 [136]. Despite most studies did not specifically evaluate *NPM1*-mutated AML, they showed that patients who might benefit more from these drug combinations are those who are naïve for hypomethylating agents or have <20% blasts and a higher pre-therapy infiltration of bone marrow by CD3+, CD4+ Teff, and CD8+ T cells [132]. The clinical trial NCT03769532 is currently evaluating the safety/efficacy of pembrolizumab plus 5-azacitidine in *NPM1*-mutated AML patients. The impact of pembrolizumab 200 mg (i.v. on day 14) has been assessed also in association with high-dose cytarabine in 37 R/R AML patients (9/37, 24%, bearing *NPM1* mutations) [137]. The overall response rate, composite CR rate and median OS were 46%, 38% and 11.1 months, respectively. Responding patients exhibited a higher percentage of progenitor exhausted TCF1+ CD8+ T cells and an increased diversity of the T-cell receptor at baseline [137].

As previously mentioned, venetoclax is very active in *NPM1*-mutated AML [70]. This effect is also contributed by the immunomodulatory effect of the drug that enhances the T-cell-mediated anti-leukemic response by increasing reactive-oxygen species (ROS) production [138] and increases the PD1+ T-effector memory cells and anti-tumor efficacy in combination with immune check-point blockade [139]. The NCT02397720 trial is assessing the combination of nivolumab, azacytidine and venetoclax in frontline and R/R AML, while the NCT04284787 trial is assessing the impact of pembrolizumab plus azacytidine and venetoclax in newly diagnosed AML patients unfit for conventional chemotherapy.

### CAR and TCR engineered T cell therapy

CAR T cells or T-cell receptor (TCR) gene therapy could be promising approaches against *NPM1*-mutated AML. Immune targeting can be distinguished into: (1) HLA-dependent therapies relying on the presentation of NPM1 neoantigen [113]; or (2) HLA-independent therapies identifying molecules differentially expressed on leukemic cells relative to normal cells (tumor-associated antigens).

Searching for HLA class I ligandome of primary AMLs, multiple ΔNPM1-derived immunogenic peptides, have been identified, including AIQDLCLAV, AIQDLCVAV, CLAVEEVSL, LAVEEVSLR, AVEEVSLRK 9-mer, and CLAVEEVSLRK 11-mer, representative of the more common *NPM1* mutation types. These peptides are able to efficiently bind to at least most common HLA types (A*02:01, A*03:01) which are often detected in the Caucasian population [111–115, 140–142]. Using yeast surface display, a human single-chain variable fragment (scFv) that specifically identifies the NPM1 mutant epitope/HLA-A2 complex but not HLA-A2 or HLA-A2 loaded with control peptides was generated and used to construct CAR T cells (Fig. 4). These engineered cells showed strong in vitro and in vivo activity against preclinical models of *NPM1*-mutated AML cells carrying NPM1-mutant/HLA-A2 complex but not against NPM1 wild-type/HLA-A2^+^ AML cells or HLA-A2 negative tumor cells [140]. More recently, memory-like NK cells armed with the same neoepitope-specific CAR showed strong activity against *NPM1*-mutated AML in absence of toxicity [143].

**Fig. 5:**
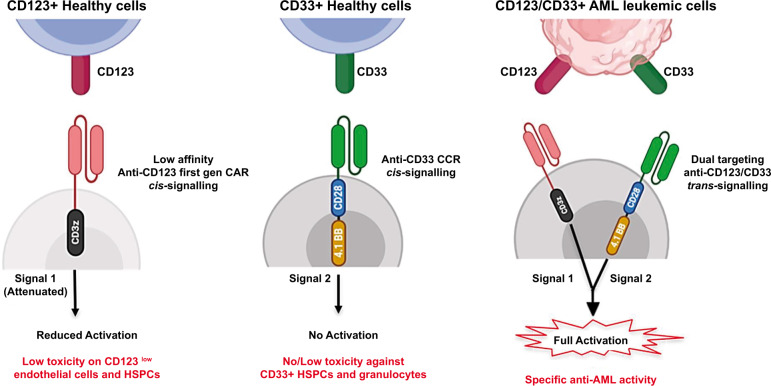
Dual CAR targeting of CD33 and CD123. The rationale of CD123/CD33 dual targeting trans-signaling strategy is to induce a full cell activation against only CD123/CD33+ leukemic cells while reducing cell stimulation against CD33+ HSPCs and CD123+ endothelial cells [144].

CD123 and CD33 are strongly expressed both in *NPM1*-mutated AML cells and healthy tissue. Thus, aiming to improve selectivity for leukemic cells while minimizing toxicity towards normal cells, a dual targeting model was exploited through Cytokine Induced Killer (CIK) cells co-expressing a first-generation low affinity anti-CD123 and an anti-CD33 as costimulatory receptor without activation signaling domains. This trans-signaling strategy could allow: i) low toxicity profile against CD123+ endothelial cells and HSPC, due to a reduced cell activation given by the suboptimal first-generation CAR signal; ii) no or low myelotoxicity against CD33+ HSPC cells, due to absence of CIK cell activation upon the sole costimulatory signal engagement; and iii) full CAR-CIK activation only against double expressing CD123+/CD33+ leukemic cells [144] (Fig. 5).

Specific T cells for HLA-A*02:01-binding CLAVEEVSL have been searched in healthy individuals using peptide-HLA tetramers. Tetramer-positive CD8+ T cells were isolated and their activity towards primary AMLs investigated. The TCR was then isolated from a clone with high anti-leukemic reactivity and its capability to specifically recognize and lyse HLA-A*02:01-positive ΔNPM1 AML demonstrated after retroviral transduction of CD8+ and CD4+ T cells [145]. Moreover, T cells transduced with TCR for HLA-A*02:01-binding CLAVEEVSL efficiently killed AML cells and prolonged OS of NSG mice engrafted with HLA-A*02:01-positive *NPM1*-mutated OCI-AML3 human cells [145] (Fig. 4). Thus, CLAVEEVSL is a neoantigen that can be efficiently targeted on AML by ΔNPM1 TCR gene transfer. While such TCR gene-engineered T-cell therapy prove to be potent and safe, it must match the TCR haplotype restriction to HLA-A*02:01-positive patients, representing around 40% of Caucasian population. Moreover, HLA class I downregulation or loss of neoantigen expression could be a possible mechanism of immune escape from *NPM1*-mutated TCR gene-engineered T-cell therapy.

DLI using T cells derived from healthy donors and specifically directed against the NPM1-mutated neoantigen with the aim to elicit graft-versus-leukemia may be a therapeutic option in patients experiencing molecular relapse following allogeneic HSCT. Eliciting endogenous immune responses through vaccination with NPM1 neoantigens is unlikely to be effective in patients with high-burden newly diagnosed or relapsed AML but could be of benefit, possibly in combination with immune check-point inhibitors, to treat pre-emptively *NPM1*-mutated AML with persistent *NPM1* transcripts or in molecular relapse.

## Future perspectives

Molecular mechanisms underlying *NPM1*-mutated AML are still poorly understood. Endogenous tagging of wild-type and mutated *NPM1* proteins followed by mass spectrometry may unravel their interactions with other partners and functions in the cytoplasm. How NPM1 mutants deregulate the HOX program remains also to be better defined. Moreover, all current experimental data are based upon the analysis of OCI-AML3 and IMS-M2 human AML cell lines that both carry the most frequent *NPM1* mutation (i.e., mutation A). Whether similar results can be extended also to the rarer *NPM1* variants remains to be determined. Clarifying these issues may lead to the development of new targeted therapeutic strategies.

Unfit *NPM1*-mutated AML patients relapsing after venetoclax-based regimens represent a medical need. The mechanisms of resistance to venetoclax and its use in combination with other drugs to prevent relapse should be better investigated. Menin inhibitors are emerging as the most promising agents for targeted therapy of *NPM1*-mutated AML. The ongoing trials will tell us which is the real impact of these compounds in *NPM1*-mutated AML and suggest which are the best combinations to maximize the clinical benefit. Menin inhibitors, alone or in combination with venetoclax or other agents, could be incorporated in the treatment algorithm, as that shown in Fig. 3A, to reduce or eradicate *NPM1*-related MRD, possibly as bridge to allo-HSCT, in eligible patients. Moreover, menin and XPO1 inhibitors have the potential to be used alone or in combination (e.g., with FLT3 inhibitors), at initial diagnosis, especially in patients who are older or unfit for intensive chemotherapy.

**Fig. 6:**
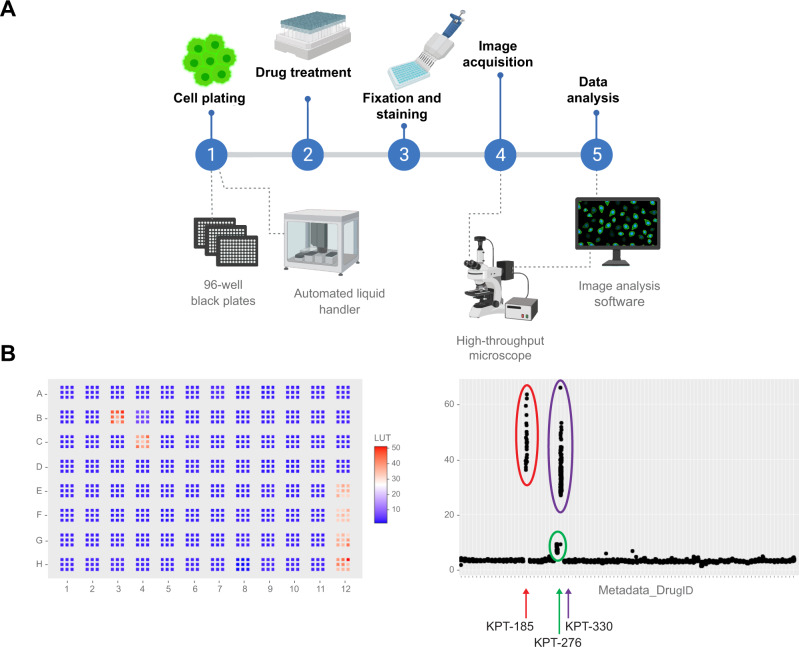
High-throughput screening for novel drugs in *NPM1*-mutated AML. **A** Workflow of microscopy-based screening strategy (created with BioRender.com). **B** Example of 96 well plate subjected to image and data processing (generated with ShinyHTM software). Arrows indicate results obtained with XPO1 inhibitors (KPT-185, KPT-276 and KPT-330). The higher number of points for selinexor (KPT-330) results from its use as a positive control.

Identifying novel drugs for *NPM1*-mutated AML using synthetic lethality approaches are under way [146]. High-throughput screening technology [147] allows to screen a large number of compound libraries at a rate that may exceed a few thousand compounds per day or per week [148, 149]. Searching for new molecules able to re-localize or reduce the expression of the NPM1 mutated protein, we have established a microscopy-based screening strategy suitable to analyze hundreds selected drugs and compounds using high-throughput microscope technique and image analysis (Fig. 6). Among other compounds, we identified inhibitors with known re-localizing activity on NPM1 mutated protein, thus confirming the value of our experimental strategy (unpublished data).
